# Increased Incidence of Pancreatic Steatosis Detected Using Computed Tomography at Initial Diagnosis of Coronavirus Disease 2019

**DOI:** 10.5152/tjg.2023.22471

**Published:** 2023-03-01

**Authors:** Ayşegül Öz, Seray Akçalar

**Affiliations:** 1 Lamed Medical Imaging Center, İzmir, Turkey; 2 Department of Radiology, Kent Health Group, İzmir, Turkey

**Keywords:** Pancreatic steatosis, non-alcoholic fatty pancreas, COVID-19, computed tomography

## Abstract

**Background:**

It is known that hepatic steatosis, diabetes, obesity, and metabolic syndrome are poor prognostic criteria for coronavirus disease 2019. Closely associated with these factors, pancreatic steatosis has yet to be clarified regarding its incidence in patients with coronavirus disease 2019 and its effect on prognosis. This study aimed to compare the incidence of pancreatic steatosis detected in non-contrast chest computed tomography examinations of patients with coronavirus disease 2019 pneumonia at the time of diagnosis with that of the general population.

**Methods:**

In the present retrospective study, which included 399 patients, densities of 5 different regions of the pancreas and 4 different regions of the spleen were measured, and the mean value of the measured densities was obtained. The difference between the mean pancreatic attenuation and splenic attenuation was defined as pancreatic steatosis if pancreatic attenuation–splenic attenuation ≤−5.

**Results:**

The median pancreatic density in patients with coronavirus disease 2019 was significantly lower than in those who tested negative (*P* = .034). In patients who were coronavirus disease 2019 positive, the incidence of pancreatic steatosis was statistically significantly higher (54.3% vs. 43.0%, *P* = .031).

**Conclusions:**

According to the non-contrast chest computed tomography examination of the patients with coronavirus disease 2019 performed at the time of admission, the incidence of pancreatic steatosis was higher than that of the normal population of a similar age group. Given that patients with pancreatic steatosis and the accompanying metabolic syndrome are more prone to inflammation, the findings suggest that these patients underwent more chest computed tomography examinations at the time of diagnosis. Therefore, pancreatic steatosis may be a poor prognostic factor in coronavirus disease 2019.

**Main Points**

- Pancreatic steatosis (PS) is related to obesity, metabolic syndrome, and hepatic fat content.
- Metabolic syndrome-related risk factors such as diabetes, hypertension, cardiovascular disease, and obesity are associated with severe prognosis and high mortality in coronavirus disease 2019 (COVID-19).
- The incidence of PS is significantly higher in patients with a positive COVID-19 status (54.3% vs. 43.0%).
- Median pancreatic density values are significantly lower in patients with a positive COVID-19 status (38 HU vs. 40 HU).
- Quantitative posttreatment measurement of PS would be useful for monitoring PS for its impact on mortality and morbidity in COVID-19.

## Introduction

Coronavirus disease 2019 (COVID-19) is a rapidly spreading infectious disease that was recognized as a global pandemic by the World Health Organization (WHO) in March 2020.^1^ According to the data made available by the WHO, as of March 2022, approximately 456 million people worldwide were infected and approximately 6 million died (https://covid19.who.int/). The first case of the disease was reported in Wuhan, China, in December 2019. The disease is caused by severe acute respiratory syndrome coronavirus 2 (SARS-CoV-2).^2^ This viral infection is transmitted via the respiratory tract and droplets can be asymptomatic or mildly symptomatic and can cause severe pneumonia that requires intensive care hospitalization and mechanical ventilation, especially in adults, and multiorgan dysfunction and mortality in severe cases.^3^ As seen in patients with acute respiratory distress syndrome, cytokine storms caused by increased levels of inflammatory molecules and proinflammatory cytokines lead to increased severity of the disease, multiorgan failure, and sepsis.^4^ In various studies published in the literature, risk factors, such as diabetes, hypertension, cardiovascular diseases, and obesity, related to metabolic syndrome (MetS), are associated with severe prognosis and high mortality in COVID-19.^5,6^ Non-alcoholic fatty liver disease or hepatic steatosis (HS) is the hepatic manifestation of MetS. The risk of severe COVID-19 infection is thought to be potentially associated with impaired liver functions due to HS.^7^ Pancreatic fat has a similar pathogenesis to that of HS, and obesity is closely related to diabetes.^8^ Although there are many publications in the literature investigating metabolic risk factors of COVID-19, very few studies have proved a relationship between pancreatic steatosis (PS) and COVID-19. The current study aimed to investigate whether the study group of patients who were diagnosed as having COVID-19 and the control group, which tested negative for COVID-19, differed in terms of PS.

## Materials and Methods

### Study Population

Our study was designed as a retrospective case-control study. The study included 234 patients in the study group, who were admitted to pandemic clinic and emergency department of Kent Health Group Hospital, İzmir with flu-like symptoms, underwent chest computed tomography (CT) examinations, and tested positive in reverse-transcriptase polymerase chain reaction (RT-PCR) tests. A total of 227 patients who tested negative in the RT-PCR test, who were followed up for nodules with chest CT, or who were liver or kidney donors and had a chest CT before surgery were included in the control group. The entire group of patients consisted of symptomatic patients who tested positive for COVID-19 in the RT-PCR test and asymptomatic patients who tested negative in the RT-PCR test. The control group included only asymptomatic patients who tested negative in the RT-PCR test to ensure a COVID-19-negative control group because patients with early false-negative RT-PCR results and parenchymal findings on chest CT could cause biased assessments. Among all the patients who tested negative in the RT-PCR test, patients with incidental parenchymal findings in the typical, atypical, and indeterminate categories defined in the “Reporting Criteria of Chest CT Findings Related to COVID-19” by the Radiological Society of North America were excluded from the study. Only patients with negative parenchymal findings on chest CT were included in the control group.^9^

Of all the patients in both groups, 62 patients with motion artifacts, no non-contrast CT, a history of splenectomy, acute-chronic pancreatitis, a history of pancreatic surgery, a history of malignancy in the pancreas, parenchymal calcifications or ductal dilatation, and a partial pancreatic examination were excluded from the study. Our study comprised 399 patients divided into 2 groups: the cohort group (199 patients who tested positive for RT-PCR) and the case-control group (200 patients who tested negative for RT-PCR). The approval for the study was granted by the Katip Çelebi University Ethics Committee (24.03.2022/0153), and all procedures were applied in accordance with the Helsinki Declaration. Informed consent was waived because of the retrospective nature of the study.

### Computed Tomography procedure

All CT examinations were performed using a Toshiba Aquilion 64-slice scanner (Toshiba Medical Systems, Irvine, Calif, USA). The main scanning parameters were as follows: collimation 64 × 0.5, 1.25-mm section thickness, 0.8-mm reconstruction interval, 120 KVp, mAs dependent on automatic modulation of the dose radiation, and matrix size 512 × 512. The field of view was 250-300 mm. The lung and abdomen settings were adjusted to a length of 600 and 400 Hounsfield units (HU) and a width of 1500 and 40 HU, respectively.

### Computed Tomography Image Interpretation

All images were evaluated by 2 radiologists with 13 years (A.O.) and 5 years (S.A.) of experience in abdominal and chest radiology, and the final decision was made by mutual consensus.

**Figure 1.**
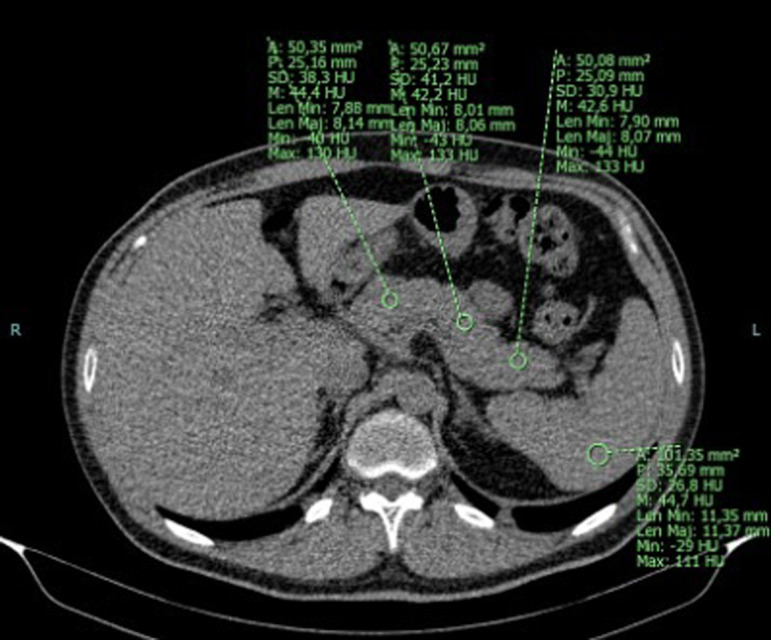
An example of the measurement of attenuation values of liver and spleen on non-enhanced computed tomography by using region of interest.

Several methods for defining CT-based investigations of PS are reported in the literature.^10,11^ Quantitative values, such as the pancreas-to-spleen ratio and the difference between pancreas attenuation (PA) and spleen attenuation (SA), are well correlated with the histologic findings that predict the amount of fat in the pancreas. In our study, we used the difference between PA and SA.^12^ Non-enhanced axial CT images were measured (Figure 1). Using a region of interest (ROI) of 0.5 cm^2^, pancreatic parenchymal attenuation was measured on 5 different regions, including the head, corpus, tail, neck, and uncinate process, and an average value was obtained. Splenic parenchymal attenuation was measured on 4 different regions using an ROI of 1 cm^2^, and a mean value of the measured attenuation was obtained. The periphery of the pancreas was avoided to prevent the possible effect of vascular structures and partial volume on the measurements. The difference between the mean PA and SA was used to evaluate PS. Pancreatic steatosis was defined as PA-SA ≤−5 (fatty pancreas), whereas PA-SA >−5 HU was defined as non-fatty pancreas.

### Statistical Analysis

To provide continuous data with normal distribution for descriptive statistical analysis, mean ± standard deviation was used. Median with minimum-maximum values was used for continuous variables without normal distribution. Numbers and percentages were used for categorical variables. The Shapiro–Wilk, Kolmogorov–Smirnov, and Anderson–Darling tests were used to analyze the normal distribution of the numerical variables.

For variables without normal distribution (age, pancreatic density, splenic density, difference between pancreatic and splenic densities, and the frequency of PS), the Mann–Whitney *U*-test was used to compare 2 independent groups depending on their COVID-19 or PS status.

Pearson’s chi-square and Fisher’s exact tests were used to compare differences between categorical variables (sex distribution and PS) and between the patient groups based on COVID-19 or PS status.

Univariate logistic regression analyses were performed to analyze the factors impacting the COVID-19 status.

For statistical analysis, “Jamovi project (2022),” Jamovi (Version 2.2.5.0) (Computer Software) (Retrieved from https://www.jamovi.org) and JASP (Version 0.16) (Retrieved from https://jasp-stats.org) were used. In all statistical analyses, the significance level (*P*-value) was determined at .05.

## Results

There were 399 patients in the study group, and 49.9% tested positive for COVID-19. The mean age was 54.3 ± 14.1 years. The male-to-female ratio was 1.5. The measured median values of the pancreatic and splenic densities were 39.0 and 45.0 HU, respectively. Pancreatic steatosis was found in 194 patients (48.6%) based on the difference between the mean pancreatic and splenic densities (Table 1).

**Figure 2.**
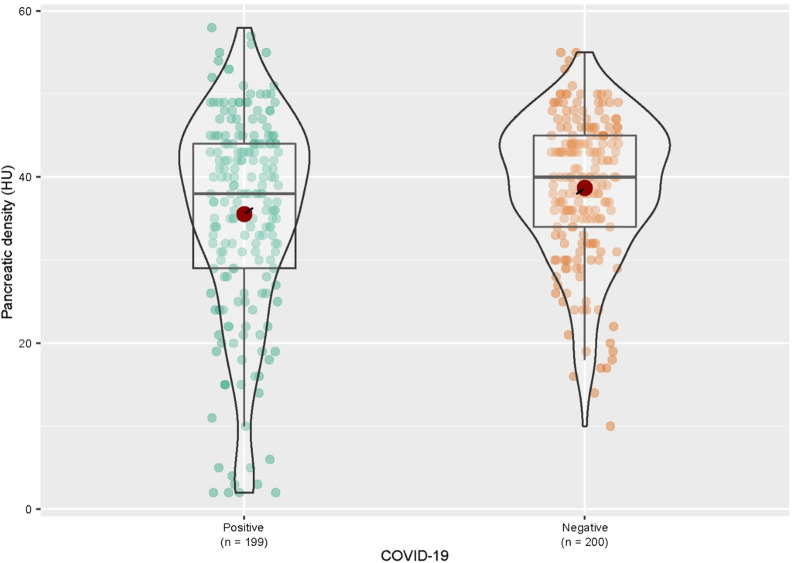
Graphic representation of the pancreatic density (HU) in patients according to their COVID-19 status. COVID-19, coronavirus disease 2019; HU, Hounsfield unit.

**Figure 3.**
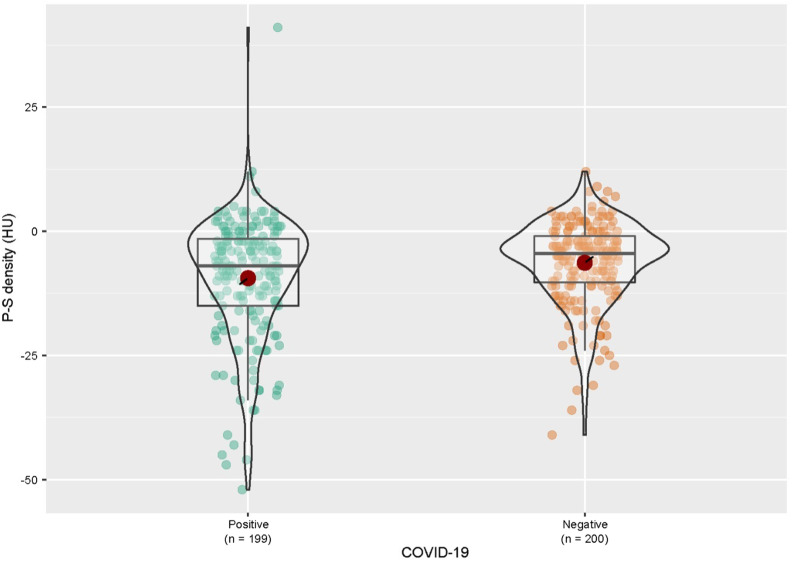
Graphic representation of the P-S density (HU) in patients according to their COVID-19 status. COVID-19, coronavirus disease 2019; HU, Hounsfield unit; P, pancreatic; S, splenic.

The groups based on COVID-19 status were similar in demographic characteristics (*P* > .05) (Table 2). Patients with a positive COVID-19 status had a significantly lower median pancreatic density than those with a negative status (*P* = .034) (Figures 2 and 3).

The incidence of PS was significantly higher in patients with a positive COVID-19 status (54.3% vs. 43.0%, *P* = .031).

The patients with PS were significantly older than those without PS (*P* > .001) (Table 3). The sex distribution was similar between the groups (*P* = .164).

**Figure 4.**
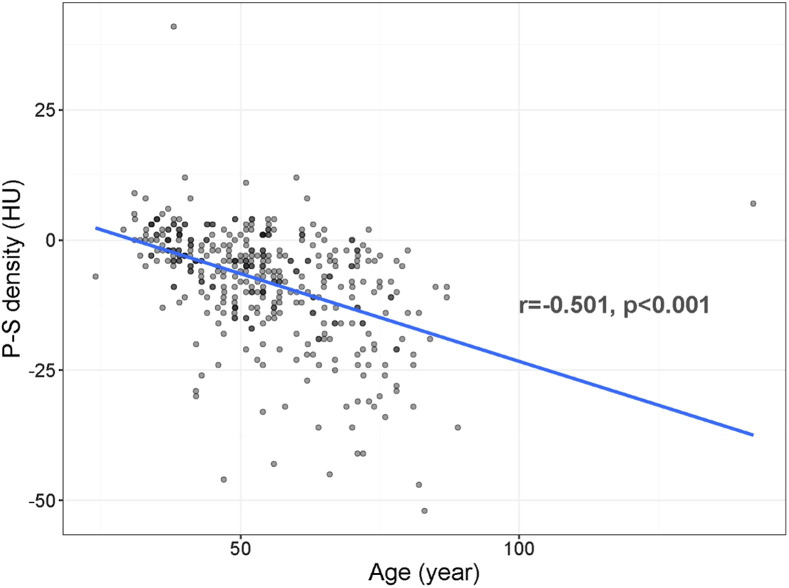
Correlation between the P-S density (HU) and age (years). HU, Hounsfield unit; P, pancreatic; S, splenic.

We detected significant negative correlations between the age of the patients and their numeric HU values of P-S densities (Table 4). The lowest values were more frequently seen in older patients (Figure 4).

A negative correlation was observed for all patients in the study group (*r* = −0.501, *P* < .001). The negative correlations between the age of the patients and their HU values of P-S densities were also significant for the patients with a positive COVID-19 status (*r* = −0.464, *P* < .001) and with PS (*r* = −0.367, *P* < .001).

Univariate logistic regression analysis showed that pancreatic density and PS were risk factors in patients who were COVID-19 positive compared with patients who were COVID-19 negative (Table 5).

## Discussion

Based on the non-contrast chest CT examination of the patients who were COVID-19 positive performed at the time of diagnosis, the rate of PS in our study was higher than in the normal asymptomatic population of a similar age group.

Coronavirus disease 2019 mortality and morbidity increase in MetS due to the abnormal release of cytokines and acute-phase reactants originating from the chronic systemic inflammation in obesity.^13,14^ Pancreatic steatosis is associated with MetS, visceral steatosis, type 2 diabetes mellitus, pancreatitis, and pancreatic neoplasia.^8,12,15,16^

Pancreatic steatosis is also classified as fatty pancreas, fatty infiltration, fatty replacement, pancreatic lipomatosis, lipomatous pseudohypertrophy, and non-alcoholic fatty pancreatic disease (NAFPD). In patients with obesity, NAFPD progresses to non-alcoholic steatopancreatitis and fibrosis may develop.^15,17^

Histopathologically, PS occurs in 2 ways: fatty infiltration and fatty replacement. Fatty infiltration defines NAFPD, where the lipid accumulates in the intracellular compartment. Obesity often causes NAFPD histopathologically. Ectopic adipocytes cause pancreatic hyperplasia and hypertrophy, thereby resulting in insulin resistance and type 2 diabetes.^18-20^ In fatty replacement, adipocytes infiltrate into acinar cells and accumulate intracellular lipids, glandular islands are irreversibly replaced by adipocytes, and pancreatic cell apoptosis and pancreatic parenchymal necrosis occur.^15,17,18^ The causes of fatty replacement could be congenital diseases, viral infections, iron loading, alcohol use, and drug-induced obstructive pancreatitis.^18^

Different radiologic methods are used for measuring PS. Ultrasound (US) can be used transabdominally or endoscopically. Both PS and pancreatic fibrosis are characterized by greater pancreatic echogenicity in US examinations.^8,18,21^ In different studies, pancreatic echogenicity is compared with hepatic–renal echogenicity, the echogenicity of the retroperitoneal adipose tissue, or the perihepatic adipose tissue to evaluate PS on US.^18,22^ The greatest limitation of US is that it is user-dependent. In transabdominal US, the pancreas cannot be viewed in approximately 1 out of 7 patients with obesity in the target patient group. In endoscopic US, the entire pancreas can be viewed, but it is an invasive method. In US examinations, the comparison of echogenicity with the surrounding adipose tissue does not provide measurable information.^15,21^ Computed tomography is preferred for its short scanning time, its noninvasive nature, and the quantitative information it provides. In our study, the most commonly used measurement method was to calculate the mean pancreatic density using multiple measurements performed on the pancreas and then compare it with the splenic density, thereby calculating the pancreatic–splenic index.^11,15,18,23^ In our study, CT was the most practical method that provides quantitative information in retrospective evaluations without the need for any additional measurement programs. Allowing the use of various techniques, magnetic resonance imaging (MRI) is the most reliable method for radiologic evaluation and PS monitoring without using ionizing radiation. Different methods, such as spectroscopy, chemical shift, multipoint Dixon measurements, and proton density fat-fraction mapping, can be used. The data closest to histologic examination is obtained on MRI.^8,15^

The pathophysiology of PS is still under investigation. Pancreatic steatosis and liver steatosis are thought to be formed by similar mechanisms. The causes and consequences of PS are not as clear as those of HS.^15^ The higher prevalence of HS in patients with COVID-19 compared with the normal population was first demonstrated in the study of Medeiros et al.^24^ In many subsequent studies, HS has been shown to increase mortality and morbidity rates in COVID-19, as well as to elevate MetS, visceral adipose tissue rate, and obesity risk.^25-28^ In a study by Guler et al.^29^ it was also shown that the severity of HS increased during COVID-19 infections. Pancreatic steatosis was associated with HS and the increased volume of visceral adipose tissue, which is the main risk factor for MetS.^17^

This information suggests that there may be several reasons explaining the higher incidence rate of PS diagnosed at the time of initial diagnosis in COVID-19 than in the normal population. First, given that patients with PS and the accompanying MetS are more prone to inflammation, these patients may have undergone more chest CT examinations at the time of admission because they were already more symptomatic than the normal population who had COVID-19. Starting from the time of diagnosis, PS may be a poor prognostic factor in COVID-19.

Second, the novel coronavirus SARS-CoV-2 can cause PS just like Reovirus, which is known to cause PS.^8^

Our study had some limitations. The patients in the 2 groups that tested either positive or negative for COVID-19 had similar age ranges and sex distribution in our study, but we excluded some other risk factors for PS, such as body mass index, alcohol use, and congenital diseases, while evaluating both groups, which constitutes the greatest limitation of the study. Also, ectopic steatosis may be overlooked in CT because it is not distributed homogeneously throughout the entire pancreas. Intracellular and extracellular steatosis cannot be distinguished on CT. The use of ionizing radiation is also another disadvantage.^15^

In our study, according to the chest CT examination of the patients who were COVID-19 positive performed at the time of initial diagnosis, the rate of PS was higher than in the normal population of a similar age group. This finding may be related to the association of PS with MetS, which has been better understood recently, and the known high mortality and morbidity rates of COVID-19 in patients with MetS. In chest CT examinations of patients with suspected COVID-19, PS can also be easily evaluated simultaneously on radiologic CT images. The presence of PS can be integrated into the radiology report in patients with COVID-19-positive results in PCR or patients with radiologic pulmonary involvement. To identify the chronic effects of the novel SARS-CoV-2 virus on the pancreas, a quantitative posttreatment measurement of PS would be useful for monitoring PS for its impact on mortality and morbidity in COVID-19 and determining whether the virus is involved in the etiology of PS, as well as comparing the findings from these post-treatment measurements with pre-COVID-19 CT examinations of these patients, if any.

## Figures and Tables

**Table 1.** Demographic and Clinical Characteristics of the Study Groups

	Overall (n = 399)
COVID-19 status^†^	
Positive	199 (49.9)
Negative	200 (50.1)
Age (year)^‡,§^	54.3 ± 14.1
	53.0 (43.0-64.0)
Sex^†^	
Male	240 (60.2)
Female	159 (39.8)
Pancreatic density (HU)^§^	39.0 (2.0-58.0)
Splenic density (HU)^§^	45.0 (0.0-60.0)
Pancreatic steatosis^†^	
Absence (P-S >−5 HU)	205 (51.4)
Presence (P-S ≤−5 HU)	194 (48.6)

^†^n (%); ^‡^Mean ± standard deviation.; ^§^Median (min-max).

HU, Hounsfield unit; P, pancreatic; S: splenic.

**Table 2.** Comparison of the COVID-19 Positive and Negative Patients Considering the Demographic and Clinical Characteristics

	COVID-19 Status		*P*
	Positive (n = 199)	Negative (n = 200)	
Age (year)^§^	54.0 [43.0, 67.0]	52.0 [43.0. 62.0]	.166*
Sex^†^			
Male	119 (59.8)	121 (60.5)	.967**
Female	80 (40.2)	79 (39.5)	
Pancreatic density (HU)^§^	38.0 [2.0 – 58.0]	40.0 [10.0 – 55.0]	.034*
Splenic density (HU)^§^	46.0 [0.0 – 60.0]	45.0 [29.0 – 55.0]	.586*
Pancreatic steatosis^†^			
Absence (P-S > −5 HU)	91 (45.7)	114 (57.0)	.031**
Presence (P-S ≤ −5 HU)	108 (54.3)	86 (43.0)	

^†^n (%), ^§^median [min-max]; *Mann-Whitney *U* test; **Pearson Chi-Square or Fisher’s Exact test.

HU, Hounsfield unit; P, pancreatic, S, splenic.

**Table 3.** Demographic Characteristics of the Patients with and without Pancreatic Steatosis

	Pancreatic Steatosis		*P*
	Present (n = 194)	Absent (n = 205)	
Age (year)^§^	57.5 (24.0-89.0)	48.0 (29.0-142.0)	<.001 ^*^
Sex^†^			
Male	124 (63.9)	116 (56.6)	.164^**^
Female	70 (36.1)	89 (43.4)	

^†^n (%); ^§^Median (min-max); ^*^Mann–Whitney *U* test; ^**^Pearson’s chi-square or Fisher’s exact test. *P* < .05 was statistically significant.

**Table 4.** Correlation of Age with the Difference Between the Pancreatic and Splenic Densities (HU) in Different Patient Groups

	Overall		Patients with COVID-19 Positivity		Patients with Pancreatic Steatosis	
	*r*	*P*	*r*	*P*	*r*	*P*
Age	−0.501	**<.001**	−0.464	**<.001**	−0.367	**<.001**

COVID-19, coronavirus disease 2019; HU, Hounsfield unit.

Spearman’s rho correlation coefficient. *P* < .05 was statistically significant.

**Table 5.** Univariate Logistic Regression Analysis of the Risk Factors for Pancreatic Steatosis and COVID-19

Dependent Parameter: COVID-19 Positivity	Univariate Analysis (OR, 95% CI)
Age	0.99 (0.97-1.00, *P* = .120)
Pancreatic density	1.03 (1.01-1.05, ***P*** ** = .004**)
Pancreatic steatosis	0.64 (0.43-0.94, ***P*** ** = .025**)

COVID-19, coronavirus disease 2019; OR, odds ratio; P, pancreatic; S, splenic. *P* < .05 was statistically significant.
